# PRL-3 activates mTORC1 in Cancer Progression

**DOI:** 10.1038/srep17046

**Published:** 2015-11-24

**Authors:** Zu Ye, Abdul Qader Omer Al-aidaroos, Jung Eun Park, Hiu Fung Yuen, Shu Dong Zhang, Abhishek Gupta, Youbin Lin, Han-Ming Shen, Qi Zeng

**Affiliations:** 1 Institute of Molecular and Cell Biology, A*STAR (Agency for Science, Technology and Research), Republic of Singapore; 2 Department of Physiology, Yong Loo Lin School of Medicine, National University of Singapore, Republic of Singapore; 3 Centre for Cancer Research and Cell Biology, Queen’s University Belfast, Northern Ireland, UK; 4 Department of Biochemistry, Yong Loo Lin School of Medicine, National University of Singapore, Republic of Singapore

## Abstract

PRL-3, a metastasis-associated phosphatase, is known to exert its oncogenic functions through activation of PI3K/Akt, which is a key regulator of the rapamycin-sensitive mTOR complex 1 (mTORC1), but a coherent link between PRL-3 and activation of mTOR has not yet been formally demonstrated. We report a positive correlation between PRL-3 expression and mTOR phospho-activation in clinical tumour samples and mouse models of cancer and demonstrate that PRL-3 increased downstream signalling to the mTOR substrates, p70S6K and 4E-BP1, by increasing PI3K/Akt-mediated activation of Rheb-GTP via TSC2 suppression. We also show that PRL-3 increases mTOR translocation to lysosomes via increased mTOR binding affinity to Rag GTPases in an Akt-independent manner, demonstrating a previously undescribed mechanism of action for PRL-3. PRL-3 also enhanced matrix metalloproteinase-2 secretion and cellular invasiveness via activation of mTOR, attributes which were sensitive to rapamycin treatment. The downstream effects of PRL-3 were maintained even under conditions of environmental stress, suggesting that PRL-3 provides a strategic survival advantage to tumour cells via its effects on mTOR.

The mechanistic target of rapamycin (mTOR) is a critical regulator of cell growth in response to a variety of stimuli, including growth factors and nutrients^1^. mTOR integrates signals from receptor tyrosine kinase (RTK)-mediated signalling pathways, such as phosphatidylinositol 3′ -kinase (PI3K)/Akt or extracellular signal-related kinases (ERK), and activates downstream targets to modulate cell growth or cellular metabolism^1^. Consistent with its central role in controlling cell growth, the mTOR signalling pathway is often hyperactivated in a broad spectrum of human cancers and metabolic diseases^2^. mTOR forms two multiprotein complexes called mTOR complex 1 (mTORC1) and mTOR complex 2 (mTORC2), which can be distinguished by their specific binding partners, raptor and rictor, respectively, as well as their sensitivity to rapamycin: mTORC1 is sensitive and mTORC2 is insensitive^3^,^4^. As a master regulator of protein synthesis, mTORC1 directly phosphorylates the translational regulators, eukaryotic translation initiation factor 4E (eIF4E)-binding protein 1 (4E-BP1) and p70 S6 kinase (p70S6K). Whereas 4E-BP1 phosphorylation blocks its inhibitory binding to eIF4E and allows for m^7^GTP cap-dependent translation to proceed, p70S6K phosphorylation promotes the formation of translation initiation complexes and enhances mRNA translation^5^.

Canonical mTOR activation depends on mitogen-driven signalling through PI3K/Akt, although non-Akt-dependent activation through the Ras/MEK/ERK pathway has also been described^6^. The effector kinases of these pathways directly phosphorylate a heterotrimeric complex consisting of tuberous sclerosis 1 (TSC1), TSC2 and TBC1D7, which collectively function as a GTPase-activating protein (GAP) for the Rheb (Ras homolog enriched in brain) GTPase^7^,^8^. Upon amino acid or serum starvation, TSC1/2 relocalizes to lysosomes where it induces the conversion of Rheb into its inactive GDP-bound state^9^,^10^. This results in mTORC1 inhibition, as it is GTP-bound Rheb which directly interacts with mTORC1 and strongly stimulates its kinase activity. Furthermore, to be physically activated by GTP-loaded Rheb, mTORC1 must also translocate to cellular endomembranes where Rheb is located^11^. The Rag GTPases, members of the Ras family of GTP-binding proteins, are essential for this^12^. Mechanistically, Ragulator, a Rag guanine nucleotide exchange factor, responds to the presence of amino acids by promoting the loading of RagA/B with GTP, thereby enabling Rag heterodimers to interact with the raptor component of mTORC1 and recruit mTORC1 to lysosomes^13^,^14^. Thus, full mTORC1 activation is a two-pronged process, requiring growth factor signalling (via PI3K/Akt) to activate Rheb, and mTORC1 translocation to Rheb-resident endomembranes, particularly lysosomes^11^.

Phosphatase of regenerating liver 3 (PRL-3), also known as PTP4A3, is a metastasis-associated protein, whose expression positively correlates with advanced cancer stages^15^,^16^. Through the activation of upstream RTKs, PRL-3 enhances cell growth and survival through multiple oncogenic effector pathways, including PI3K/Akt, Ras/MAPK, and SRC^17^,^18^,^19^. PRL-3 has also been shown to increase the activation of Akt by the concomitant downregulation in protein expression levels of the main negative regulator of PI3K/Akt activity, the phosphatase and tensin homolog (PTEN) phosphatase^20^. Analysis of cancer patient samples reveal a high frequency of PRL-3 expression in tumours but not in paired normal tissues of patients^21^, highlighting the significance of PRL-3 as a marker of poor prognosis in multiple cancer types^16^,^22^. Therefore, the understanding of the cellular roles of PRL-3 has emerged as a new frontier in cancer research.

Given that cells overexpressing PRL-3 exhibit shared characteristics with cells possessing hyperactive PI3K/Akt/mTOR signalling, including enhanced cell proliferation, survival, and motility, we hypothesized that PRL-3 might potentially play a role in mTOR regulation, as well in cancer progression. PRL-3 was shown previously to promote autophagy in ovarian cancers^23^, a phenomenon typically inhibited by mTOR activity^24^. However, this observation was later shown to be rapamycin-insensitive, discounting a role for mTORC1 as a proxy for PRL-3-driven autophagy. Herein, to conclusively address the role of mTOR in PRL-3 signalling, we thoroughly examined the correlation of PRL-3 with mTOR activity in: 1) human clinical cancer samples, 2) a mouse model of spontaneous mammary tumour development, and 3) a panel of transgenic tumour cell lines. Collectively, our results uncover a role for oncogenic PRL-3 signalling via mTORC1 both *in vivo* and *in vitro*.

## Results

### PRL-3 expression correlates with mTOR activity *in vivo* and *in vitro*

**Figure 1:**
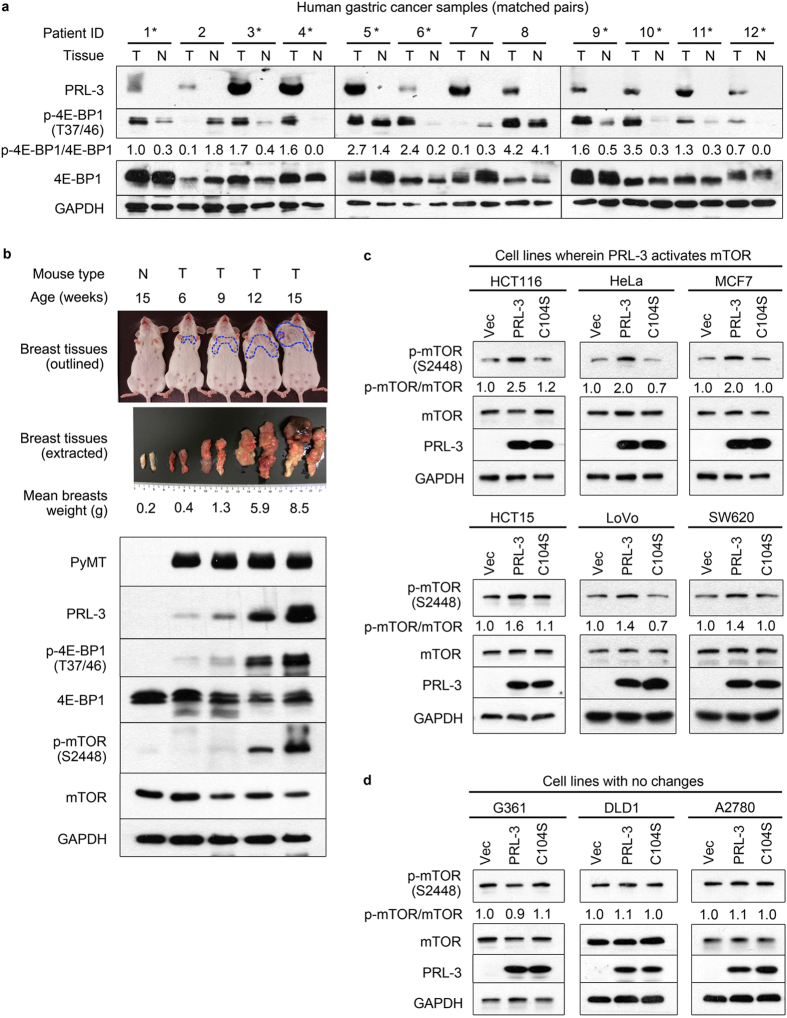
PRL-3 expression positively correlates with mTOR activity *in vivo* and *in vitro*. (**a**) Immunoblotting of matched pairs of tumour-normal tissues from PRL-3-positive cancer patients. The ratio of phosphorylated/total 4E-BP1 band densities were calculated and normalized to the phosphorylated/total 4E-BP1 ratio in lane 1. *Asterisks*, patient tumour samples wherein 4E-BP1 hyperphosphorylation correlates with PRL-3 expression. (**b**) Immunoblotting of normal and MMTV-PyMT mammary tissues over the course of spontaneous tumour development. *Top panel*, images of normal mice (N) or transgenic MMTV-PyMT mice (T) between the ages of 6 to 15 weeks. *Middle panel*, representative images of excised breast tissues from mice. All images were photographed by Thura Min. *Blue dashed lines*, gross size of palpable mammary tumours in MMTV-PyMT mice. *Lower panels*, correlation between PRL-3 expression and phosphorylation of 4E-BP1 and mTOR. (**c–d**) Overexpression of EGFP vector only (Vec), EGFP-tagged wild-type PRL-3 (PRL-3) or EGFP-tagged catalytic-inactive PRL-3 (PRL-3 C104S) in a panel of 9 human cancer cell lines. PRL-3 upregulates mTOR phosphorylation in (**c**) HCT116, HeLa, MCF7, HCT15, LOVO, and SW620 cells, but not in (**d**) G361, DLD1 or A2780 cells. GAPDH served as a loading control. The ratio of phosphorylated/total mTOR band densities were calculated and normalized to the phosphorylated/total mTOR ratio in lane 1. Full immunoblots for the cropped images presented here are provided in the Supplementary Information.

To investigate the relationship of PRL-3 with mTOR activity, we analysed 12 sets of PRL-3-positive tumours and matched normal tissue samples from gastric cancer patients for protein expression levels of PRL-3 and phosphorylation status of Thr37/46 of 4E-BP1, a direct substrate/effector of mTOR and indicator of mTOR oncogenic activity^25^. We found that PRL-3 protein was exclusively expressed in tumours but not in any of the matched normal samples (Fig. 1a). Notably, 9 out of 12 (75%) PRL-3-expressing tumours also expressed higher ratios of phosphorylated 4E-BP1/total 4E-BP1 than their matched PRL-3-negative normal tissue samples (asterisks, Fig. 1a). These clinical results implied a possible relationship between PRL-3 expression levels and mTOR activity in tumour tissues.

We further investigated the above finding in the spontaneous mouse mammary tumour virus (MMTV) transgenic model, which harbours the polyomavirus middle T oncoprotein (PyMT) under transcriptional control of the MMTV promoter-enhancer, resulting in the formation of palpable mammary tumours in mice as early as 6 weeks of age^26^. In the MMTV-PyMT system, PyMT is highly expressed in mammary tissues at relatively constant levels in heterozygous transgenic adult female mice (Fig. 1b). In contrast, endogenous PRL-3 protein expression increases steadily over the same period, specifically accumulating at later stages of tumour development (Fig. 1b). This increase in PRL-3 expression during mammary tumour development was closely correlated with an increase in levels of both phosphorylated 4E-BP1 and phosphorylated mTOR on Ser2448, but not total protein expression levels or either (Fig. 1b). Ser2448 of mTOR lies within the C-terminal ‘repressor domain’ of mTOR, and its phosphorylation is an important marker for activation of the mTOR/4E-BP1 pathway^27^,^28^. Collectively, our *in vivo* observations suggested the existence of a correlation between PRL-3 expression levels, mTOR activity, and mTOR activation-associated phosphorylation.

PRL-3 was shown previously to promote autophagy in ovarian cancers^23^, a phenomenon inhibited by mTOR activity^24^. To clarify the apparent discordance between the activation of both mTOR and autophagy by PRL-3, we analysed the consequences of overexpressing EGFP-PRL-3 (PRL-3), EGFP-PRL-3-C104S (C104S; catalytic-inactive mutant), or empty EGFP vector (Vec) in nine human cancer cell lines from diverse cancer types. In six out of the nine cell lines tested, we observed consistent hyperphosphorylation of mTOR upon overexpression of PRL-3 (Fig. 1c) but not the catalytic-inactive PRL-3 mutant or vector control. However, in three out of nine cell lines, no correlation was observed between PRL-3 expression and mTOR phosphorylation (Fig. 1d). Notably, this latter group included A2780 ovarian cancer cells, wherein PRL-3 was previously reported to activate autophagy^23^. Interestingly, rapamycin-mediated inhibition of mTOR failed to abolish the increase in autophagy promoted by PRL-3 in A2780 cells^23^, indicating an mTOR-independent route of autophagy activation by PRL-3 in this cell line. Taken together, our results suggest that PRL-3 overexpression in multiple cell lines results in mTOR phospho-activation in a catalytic-dependent and cell-specific manner.

### PRL-3 promotes mTOR activation under normal and stressed cellular conditions via increased Akt-TSC2 signalling

**Figure 2:**
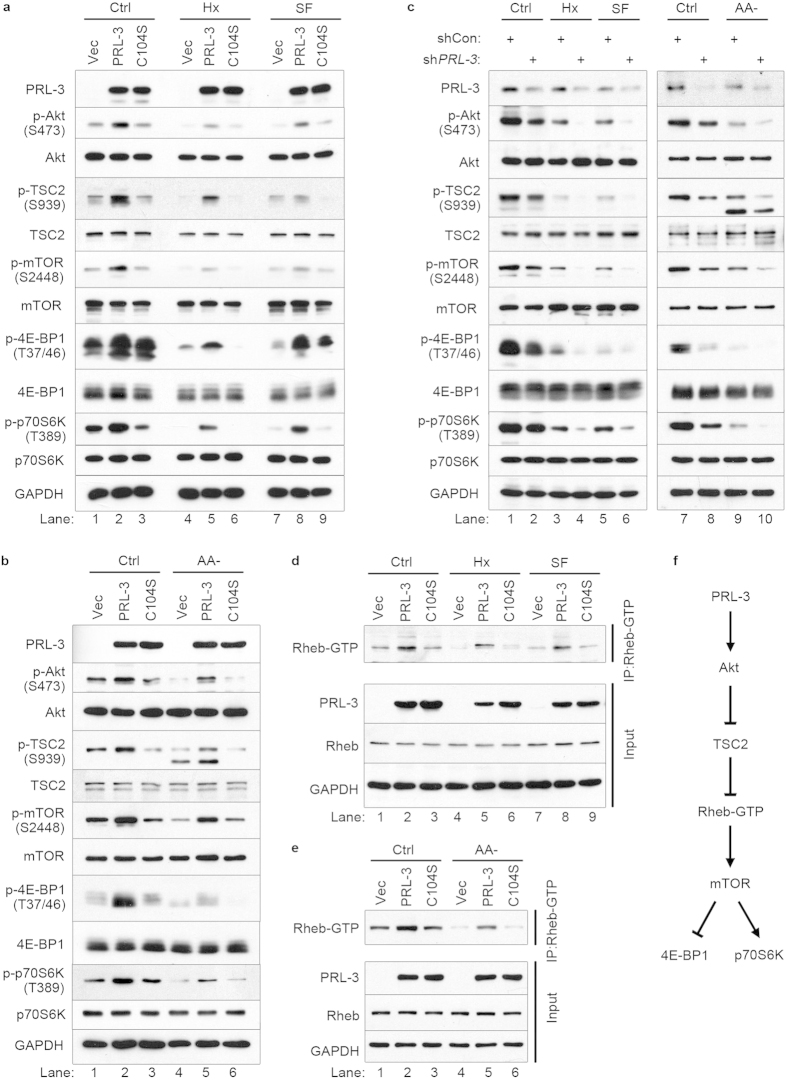
PRL-3-driven mTOR activation correlates with increased Akt-TSC2-Rheb signalling. (**a**) HCT116 cells overexpressing EGFP vector only (Vec), EGFP-tagged wild-type PRL-3 (PRL-3) or EGFP-tagged catalytic-inactive PRL-3 C104S (C104S) were cultured for 24 h in full media under normoxia (Ctrl), hypoxia (Hx), or serum-free (SF) conditions, prior to lysis and western blot analysis with the indicated antibodies. (**b**) HCT116 cells overexpressing Vec, PRL-3 or C104S were cultured for 1 h in full media under normoxia (Ctrl) or amino-acid starved (AA-) conditions, prior to analysis as in (**a**). (**c**) HCT116 cells stably expressing shRNA against PRL-3 (sh*PRL-3*) or control shRNA (shCon) were cultured and analysed as in (**a**). (**d**) Cell lysates from (**a**) were immunoprecipitated with a configuration-specific anti-Rheb-GTP antibody and analysed by immunoblotting with the indicated antibodies. (**e**) Cell lysates from (**b**) were analysed as in (**d**). (**f**) Proposed signalling pathway for PRL-3 to mTOR substrates, 4EBP1 and p70S6K. Representative full immunoblots for the cropped images presented here are provided in the Supplementary Information.

The mTOR pathway integrates multiple environmental cues to regulate translation in response to stress. Deprivation of oxygen or nutrients, particularly amino acids, results in reduced mTOR phosphorylation and inhibition of downstream effectors of protein translation, including 4E-BP1 and p70S6K^29^,^30^. mTOR activity is regulated, to a large extent, by the PI3K/Akt pathway and, as such, Akt directly phosphorylates TSC2 to relieve its inhibition of mTOR^31^,^32^. Since PRL-3 has been reported to activate PI3K/Akt signalling^33^, we first investigated if PRL-3-overexpressing HCT116 cells would display altered response to hypoxia (oxygen-deprivation) or serum starvation, two environmental stressors well-characterized to regulate mTOR activity^34^. Indeed, HCT116 cells stably overexpressing EGFP-PRL-3 (PRL-3) had higher levels of phosphorylation of Akt on Ser473, a critical activation site on this kinase^35^, under all conditions tested (Fig. 2a, lanes 2, 5 and 8). This correlated with an increase in phosphorylation and inactivation of TSC2, and a corresponding increase in phosphorylation of mTOR and its downstream substrates, 4E-BP1 and p70S6K (Fig. 2a, lanes 2, 5 and 8). Next, we investigated whether PRL-3’s ability to promote mTOR activity remained intact upon amino acid starvation, a potent inhibitory stimulus of mTORC1 activity via TSC2-mediated suppression^9^,^10^. Remarkably, relative to HCT116-Vec or HCT116-C104S cells, HCT116-PRL-3 cells displayed persistent activation of Akt/TSC2/mTOR signaling under this condition (Fig. 2b, lane 5). A similar activation of the Akt-TSC2-Rheb-mTOR-4E-BP1/p70S6K cascade was also observed in HeLa cells engineered to overexpress PRL-3 (Supplementary Fig. 1a,b). In a complimentary approach, we used small hairpin RNA (shRNA) constructs to stably deplete PRL-3 from HCT116 cells, which express endogenous PRL-3 abundantly. This resulted in reduced phosphorylation of Akt, TSC2, mTOR, and its downstream effectors, 4E-BP1 and p70S6K, under all normal and stress conditions tested (Fig. 2c, lanes 2, 4, 6, 8, 10).

To validate whether Rheb was involved in PRL-3-mediated TSC2 suppression and activation of mTOR, we used a Rheb activation assay to directly study the levels of active Rheb (Rheb-GTP) in PRL-3-overexpressing cells. Relative to HCT116-Vec or HCT116-C104S cells, Rheb-GTP levels were higher in HCT116-PRL-3 cells under basal, hypoxic, or serum starved conditions (Fig. 2d, lanes 2, 5 and 8), as well as under amino acid starvation (Fig. 2e, lane 5). Collectively, our results establish a coherent relationship between PRL-3 overexpression and increased activity of the canonical Akt/TSC2/Rheb/mTOR signaling pathway under both basal and stress conditions (Fig. 2f).

**Figure 3:**
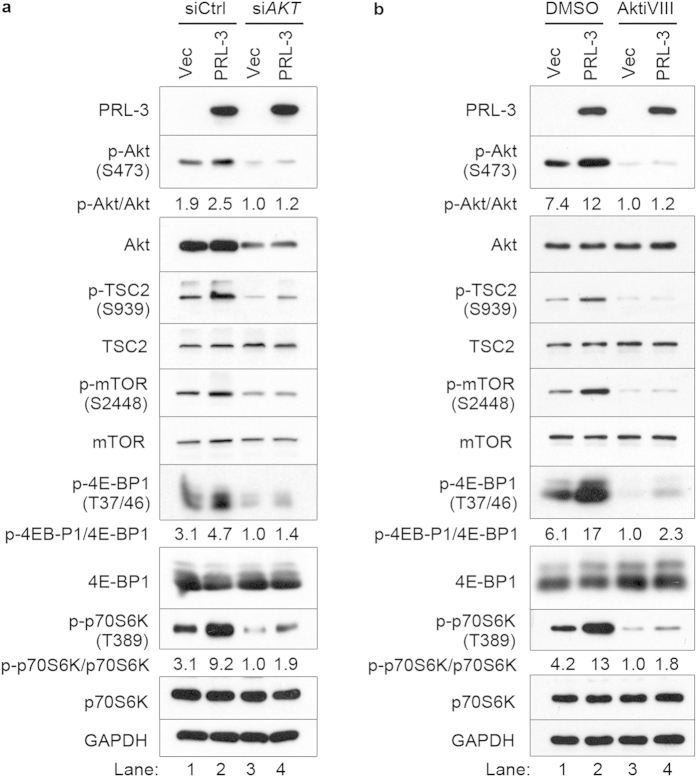
Akt activity is required for PRL-3-mediated hyperactivation of mTOR signaling. (**a**) Akt in HCT116 cells stably expressing EGFP (Vec) or EGFP-PRL-3 (PRL-3) were transiently depleted using scrambled small interfering RNA (siRNA; siCon) or AKT-targeting siRNA (si*AKT*) and cultured for 48 h before analysis of Akt-mTOR pathway activity. The ratio of phosphorylated/total band densities for Akt, 4E-BP1, and p70S6K were calculated and normalized to their cognate phosphorylated/total protein ratio in lane 3. (**b**) Akt in HCT116 Vec or PRL-3 cells was inhibited for 30 min using Akt inhibitor VIII (AktiVIII) before analysis of Akt-mTOR pathway activity as in (**a**). The ratio of phosphorylated/total band densities for Akt, 4E-BP1, and p70S6K were calculated and normalized to their cognate phosphorylated/total protein ratio in lane 3. Full immunoblots for the cropped images presented here are provided in the Supplementary Information.

Next, we sought to validate the role of Akt in PRL-3-mediated mTOR hyperactivation using two approaches: small interfering RNA (siRNA)-mediated depletion of *AKT* transcripts, or inhibition of Akt kinase using a small-molecule antagonist. siRNA-mediated *AKT* depletion reduced PRL-3-driven phosphorylation of TSC2, mTOR, and its downstream effectors, 4E-BP1 and p70S6K (Fig. 3a, lane 4). Likewise, treatment of cells with Akt inhibitor VIII (AktiVIII), a highly potent small-molecule inhibitor of Akt1/2^36^, also reduced PRL-3-induced hyperphosphorylation of TSC2, mTOR, 4E-BP1, and p70S6K (Fig. 3b, lane 4). However, we noted that HCT116-PRL-3 cells still had higher 4E-BP1 and p70S6K phosphorylation relative to HCT116-Vec cells under these treatments, suggesting that Akt inhibition did not completely abolish the ability for PRL-3 to enhance S6K and 4E-BP1 phosphorylation. Similar results were observed in HeLa cells (Supplementary Fig. 2a,b). Collectively, while these results point to a role of Akt-TSC2 signalling in PRL-3-mediated mTOR hyperphosphorylation, the elevated mTOR activity despite Akt inhibition hinted at the existence of a secondary, Akt-independent PRL-3-driven mTOR activation mechanism.

### PRL-3 promotes the relocalisation and accumulation of lysosomal mTOR in an Akt-independent, Rag GTPase-dependent manner

**Figure 4:**
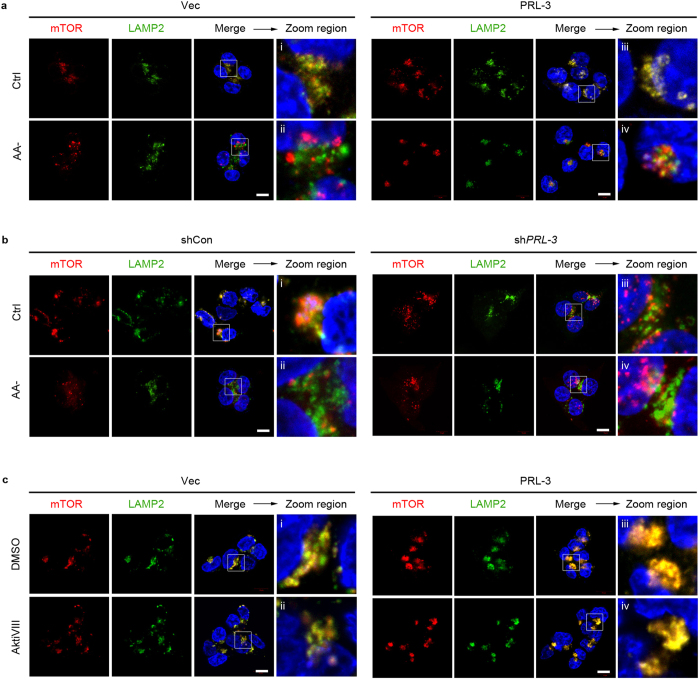
PRL-3 promotes the relocalization and accumulation of lysosomal mTOR in an Akt-independent manner. (**a**) Immunofluorescence analysis of mTOR and LAMP2 in HCT116 cells overexpressing EGFP vector only (Vec) or EGFP-tagged wild-type PRL-3 (PRL-3) cultured in full media (Ctrl) or amino-acid starvation media (AA-). Antibodies against human LAMP2 and mTOR were used. *Red*, mTOR signal; *green*, LAMP2 signal; *merge*, merged mTOR, LAMP2, and DNA (DAPI) signals. *Scale bar*, 10 μm. A zoomed area within each merged panel enables better visualize mTOR/LAMP2 colocalization. (**b**) HCT116 cells stably expressing small hairpin RNA (shRNA) against PRL-3 (sh*PRL-3*) or control shRNA (shCon) were cultured and analysed as in (**a**). *Scale bar*, 10 μm. (**c**) HCT116 Vec or PRL-3 cells were treated with DMSO or Akt Inhibitor VIII (AktiVIII) for 30 min and analyzed by dual immunfluoresence using antibodies against mTOR and LAMP2. *Red*, mTOR signal; *green*, LAMP2 *Scale bar*, 10 μm.

Despite a reduction in overall mTOR phosphorylation in cells grown under hypoxia, serum deprivation, or amino acid starvation, the PRL-3-driven increase in phosphorylation of the mTOR substrates 4E-BP1 and p70S6K under each of these stressors consistently appeared greater compared to the increase in mTOR S2448 phosphorylation itself (Fig. 2a–c, Supplementary Fig. 1a,b). This indirectly suggests that the heightened resistance conferred by PRL-3 against mTOR inactivation under limited oxygen or nutrient supply might be due to additional regulatory mechanism(s) on mTOR. In addition to canonical PI3K/Akt signalling, which regulates mTORC1 activity via TSC2-Rheb^6^, mTORC1 is also tightly regulated by changes to its localisation within the cell. In amino acid-starved cells, mTORC1 is rapidly delocalized from lysosomes, effectively inactivating this kinase^10^. Using colocalization analysis of mTOR with a lysosomal marker, LAMP2, we thus investigated if PRL-3 might activate mTOR under amino acid withdrawal by retaining lysosomal mTOR. In HCT116-Vec cells, amino acid starvation for 1 h resulted in a loss of mTOR/LAMP2 colocalization (Fig. 4a, panel ii). In contrast, mTOR/LAMP2 colocalization, albeit reduced, could still be observed in amino acid-starved HCT116-PRL-3 cells (Fig. 4a, panel iv). The ability for PRL-3 to maintain lysosomal mTOR accumulation suggested that PRL-3 overexpression might ‘mimic’ amino acid stimulation under these conditions, supporting our earlier observation of sustained mTOR signalling activity (Fig. 2b, lane 5). To confirm these results, we analyzed the mTOR/LAMP2 colocalization in HCT116 cells stably depleted of PRL-3. Depletion of endogenous PRL-3 resulted in a decrease in mTOR/LAMP2 colocalization even under basal, amino acid-replete conditions (Fig. 4b, panel iii), suggesting an important role for endogenous PRL-3 in regulating the recruitment/retention of lysosomal mTOR. To further address if enhanced PI3K/Akt signalling might account for the increased lysosomal localisation of mTOR, we treated HCT116 cells with an Akt inhibitor and analysed mTOR/LAMP2 colocalisation. No change in mTOR/LAMP2 colocalization was observed under these conditions (Fig. 4c), suggesting that these phenomenon occurred via a mechanism independent of Akt activity.

**Figure 5:**
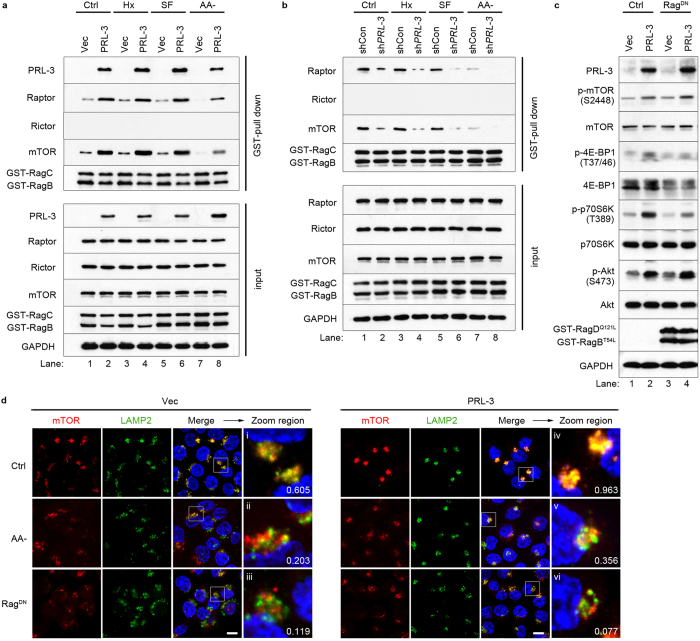
PRL-3 promotes the accumulation and activation of lysosomal mTOR via increased Rag GTPase binding. (**a**) HCT116-EGFP (Vec) or HCT116-EGFP-PRL-3 (PRL-3) cells were co-transfected with GST-RagB/C and cultured under normoxia (Nx, 24 h), hypoxia (Hx, 24 h), serum-free (SF, 24 h), or amino-acid starved (AA-, 1 h) conditions before GST pull-down and analysis by immunoblotting. *Top panel*, GST-enriched fraction; *bottom panel*, total protein input. (**b**) HCT116 cells stably expressing small hairpin RNA (shRNA) against PRL-3 (sh*PRL-3*) or control shRNA (shCon) were cultured and analysed as in (**a**). (**c**) Immunoblotting of HCT116-EGFP (Vec) and HCT116-EGFP-PRL-3 (PRL-3) cells overexpressing dominant negative RagB^T54L^-RagD^Q121L^ (Rag^DN^) or vector control (Ctrl). (**d**) HCT116-Vec or HCT116-PRL-3 cells were transfected with empty vector (Ctrl) or dominant negative RagB^T54L^-RagD^Q121L^ (Rag^DN^) for 24 h, or starved of amino acid for 1 h, before dual immunfluoresence analysis using antibodies against mTOR and LAMP2.The Manders coefficient (colocalization score) for mTOR/LAMP2 signals are given at the bottom right corners of panels i-vi. *Red*, mTOR signal; *green*, LAMP2. *Scale bar*, 10 μm. Full immunoblots for the cropped images presented here are provided in the Supplementary Information.

The Rag small GTPases have been reported to be critical regulators of mTOR lysosomal relocalisation and activation. Upon amino acid stimulation, Rag GTPases associate with mTORC1 as heterodimers, recruiting it to late endosomal and lysosomal compartments for subsequent Rheb-mediated activation^12^. Rag GTPase heterodimeric complexes (RagB-RagC) were co-expressed in either HCT116-Vec or HCT116-PRL-3 cells and binding affinities of Rag GTPases to mTOR, raptor, and rictor were examined. We found that relative to control cells, PRL-3 overexpression increased the binding affinity of Rag GTPase heterodimers to both mTOR and raptor, a component of mTORC1, under basal conditions (Fig. 5a, lane 2). Notably, no binding between Rag GTPases and rictor, a component of mTORC2, was observed in either cell line (Fig. 5a). Importantly, PRL-3-overexpressing cells maintained strong binding of Rag GTPase heterodimers to mTORC1 (mTOR and raptor) persistently under hypoxia, serum-free and amino acid-starved conditions (Fig. 5a, lanes 4, 6 and 8). In contrast, HCT116 cells depleted of PRL-3 displayed reduced Rag-mTOR-Raptor interaction under all basal and stress conditions tested (Fig. 5b, lanes 2, 4, 6 and 8). Collectively, the Rag-mTOR interaction results are in agreement with our observations showing the persistent lysosomal accumulation and elevated mTOR signalling induced by PRL-3 under basal and stress conditions.

To test if elevated Rag GTPase activity affected mTOR activity, we collected lysates from cells overexpressing dominant-negative GST-tagged RagB^T54L^-RagD^Q121L^ heterodimers^9^ (Rag^DN^) or empty vector (Ctrl) and checked phosphorylation levels of various components of the Akt-mTOR signalling pathway. Overexpression of Rag^DN^ did not rescue mTOR phosphorylation levels (Fig. 5c, lane 4), despite potently blocking the PRL-3-mediated accumulation of mTOR at LAMP2-enriched puncta (Fig. 5d, panel vi). However, a reduction in phosphorylation levels of p70S6K and 4E-BP1 was observed, suggesting suppression of mTOR kinase activity and downstream signaling by Rag^DN^ (Fig. 5c, lane 4). Notably, no changes in PRL-3-induced phosphorylation of pAkt-S473 was observed upon Rag^DN^ expression (Fig. 5c, lane 4), in agreement with our earlier data suggesting that Akt lay upstream of mTORC1. Collectively, our results show that in parallel and independent of activation of mTOR via the PI3K-Akt-TSC2-Rheb pathway, PRL-3 also specifically enhances lysosomal recruitment of mTORC1, but not mTORC2, in a Rag-dependent manner.

### PRL-3 promotes motility, invasiveness, and MMP-2 secretion in a rapamycin-sensitive manner

**Figure 6:**
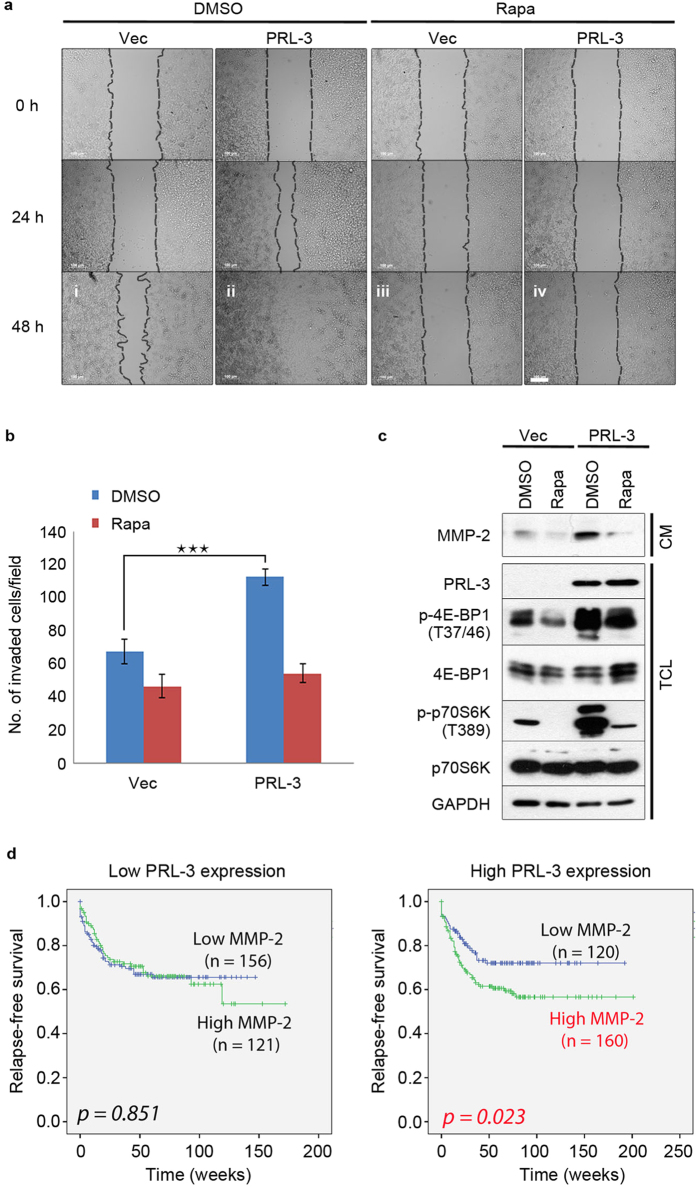
PRL-3 promotes cancer cell motility, invasiveness, and MMP-2 secretion in a rapamycin-sensitive manner. (**a**) Monolayers of HCT116-EGFP (Vec) or HCT116-EGFP-PRL-3 (PRL-3) cells were ‘wounded’ and monitored over 48 h in the presence or absence of 100 nM rapamycin (Rapa). *Dashed lines*, boundary of cell monolayers on either side of the wound. *Scale bar*, 100 μm. (**b**) The number of invaded HCT116-EGFP (Vec) or HCT116-EGFP-PRL-3 (PRL-3) cells through a basement matrix was measured by microscopy after culturing in the presence or absence of 100 nM rapamycin. Results are depicted as mean ± S.D; ***p = 5.988E-05. (**c**) The conditioned medium (CM) from HCT116-EGFP (Vec) or HCT116-EGFP-PRL-3 (PRL-3) cells after culturing in the presence or absence of 100 nM rapamycin for 24 h was harvested, concentrated and analysed by immunoblotting. *TCL*, total cell lysates from matched cell cultures. (**d**) Kaplan-Meier analysis of colorectal cancer patient cohort GSE40967 (*n* = 557) stratified by low or high PRL-3 expression. Full immunoblots for the cropped images presented here are provided in the Supplementary Information.

PRL-3 has been described to promote metastatic development of cancers by increasing both motility and invasiveness of cancer cells^33^. In a wound healing assay, PRL-3-overexpressing cells were much more motile than control cells, with complete wound closure observed by 48 h (Fig. 6a, panel i-ii). The motility of both cell lines could be effectively suppressed by rapamycin treatment (Fig. 6a, panels iii-iv), suggesting a role for mTOR signalling in PRL-3-driven motility. Besides increased motility, the ability of disseminating tumour cells to degrade the extracellular matrix (ECM) is an essential property for tumour invasion into surrounding tissues. Previously, overexpression of PRL-3 was reported to promote the invasiveness of colon cancer cells^37^ and correlate with clinical hepatocellular carcinoma invasiveness^38^. In agreement with these previous findings, overexpression of PRL-3 significantly increased invasiveness of HCT116 cells through a basement matrix relative to control cells (Fig. 6b; *p* = 5.988E-05). Importantly, rapamycin treatment suppressed invasiveness of PRL-3-overexpressing cells to similar levels as the control cells (Fig. 6b). These results suggest that mTOR is an important mediator of both PRL-3-driven motility and invasion.

Matrix metalloproteinases (MMPs) are a group of enzymes which can degrade ECM and activate a number of growth factors, thus playing important roles in tumour invasion and metastasis^39^,^40^. Notably, production of several MMPs are upregulated by increased PI3K/Akt/mTOR activity and are suppressible by rapamycin treatment^39^,^40^. Since the increased invasiveness of PRL-3-overexpressing cells could be suppressed by rapamycin, we analysed these cells in the presence or absence of rapamycin for changes in production of MMP-2, which has previously been reported to be regulated by mTOR^41^,^42^. Compared to control cells, PRL-3-overexpressing cells had higher levels of secreted MMP-2 protein in conditioned medium (Fig. 6c). Rapamycin treatment of cells also reduced the secretion of MMP-2 (Fig. 6c), suggesting that PRL-3 was mediating the upregulation of MMP-2 production via increased mTORC1 activity. Previously, PRL-3 was reported to upregulate MMP-2 production in an ERK-dependent manner^37^. Since ERK can also activate mTOR via post-translational inactivation of TSC2^43^, we treated cells with U0126, an ERK inhibitor, to study if ERK signaling might be involved in PRL-3-mediated mTOR activation. Although PRL-3 overexpression resulted in ERK1/2 activation in our system, U0126 treatment did not blunt activation of mTOR signaling downstream of PRL-3 (Supplementary Fig. 3a,b), suggesting that mTOR hyperactivation by PRL-3 occurs via an ERK-independent pathway.

**Figure 7:**
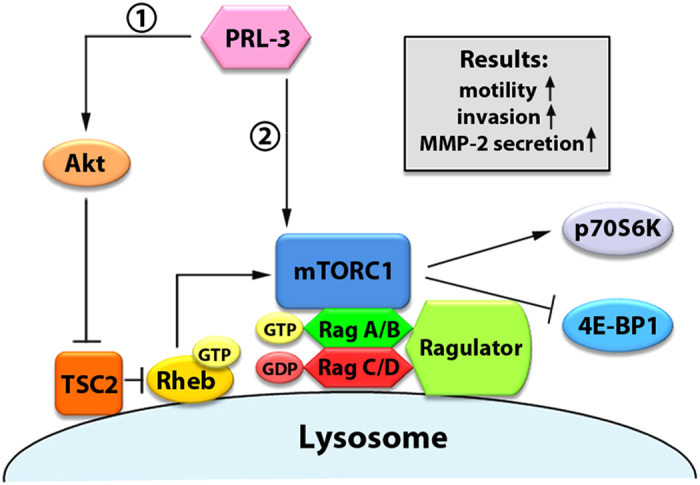
Proposed model of PRL-3 activation of mTORC1. PRL-3 activates mTORC1 activity through (1) Akt/TSC2/Rheb hyperactivation, and (2) enhanced binding to Rag GTPases and lysosomal recruitment. PRL-3-driven mTOR activation ultimately results in increased motility, invasion and MMP-2 secretion of cancer cells.

We further analysed the correlation in gene expression between PRL-3 and MMPs in a publically available clinical dataset from colon cancer patients (GSE40967, *n* = 566). Supporting our *in vitro* data, PRL-3 expression positively correlated with expression of MMP-2 (*p* = 1.287E-4) Thus, PRL-3 expression appears to correlate with an mTOR-dependent invasive phenotype characterized by the increased expression of MMP-2. Furthermore, in colon cancer patients, MMP-2 expression had prognostic value for patient survival when PRL-3 was highly expressed (*p* = 0.023; Fig. 6d, right panel), but not when PRL-3 was expressed at lower levels (*p* = 0.851; Fig. 6d, left panel), suggesting that the value of MMP-2 as a prognostic marker might be associated with PRL-3-driven mTOR hyperactivity. Herein, we propose a dual-pronged signalling model for PRL-3 activation of mTORC1 via PI3K/Akt and Rag GTPases, culminating in increased motility, invasion, and MMP-2 production by PRL-3-overexpressing cells (Fig. 7).

## Discussion

In this study, we found that PRL-3 overexpression induces an aberrant activation of mTOR kinase in cancer cells, as reflected by hyperphosphorylation of the direct substrates of mTORC1, 4E-BP1 and p70S6K. This phenomenon was more obvious under the stress conditions of oxygen, serum, or amino acid deprivation, with sustained mTORC1 activation by PRL-3 despite these growth limitations. We formally demonstrate the signalling pathway by which PRL-3 induces mTORC1 activation as via the Akt-TSC2-Rheb signalling pathway. Furthermore, we show that PRL-3 also mediates mTORC1 accumulation at lysosomes where it can be activated by Rheb. This latter function of PRL-3 is independent of Akt, and is mediated by increased binding affinity of mTOR and raptor to Rag GTPases. Functionally, PRL-3-mediated enhancement of mTORC1 signalling resulted in increased motility, invasiveness, and MMP-2 secretion. In summary, our findings reveal a novel pathway of PRL-3-mediated cancer invasiveness via mTORC1 activation.

In matched tumour-normal patient cancer samples, we observed a striking correlation between PRL-3 expression and the ratio of phosphorylated/total 4E-BP1, a *bona fide* readout of PI3K/Akt/mTOR pathway activity^44^. Supporting this *in vivo* observation, increased expression of PRL-3 during tumour progression in the MMTV-PyMT spontaneous transgenic mouse model was closely mirrored by an increase in both mTOR and 4E-BP1 phosphorylation levels. Despite previous studies reporting the association between PyMT expression and activation of PI3K/Akt signaling^45^,^46^,^47^, our results herein indicate that the increase in both phosphorylated 4E-BP1 and mTOR levels over the course of PyMT mice development more closely mirrored that of PRL-3 expression rather than PyMT strictly. These observations, generated from completely different systems, led us to hypothesize about the co-existence of a relationship between PRL-3 and mTOR. We validated *in vitro* that PRL-3 overexpression could induce mTOR phosphorylation in six out of the nine human cancer cell lines tested. Notably, this appeared independent of activating PIK3CA mutations, since mTOR activation was observed in cells harbouring both wild type (SW620, LoVo, HeLa) or mutant (HCT116, MCF7, HCT15) PIK3CA^48^, suggesting that PRL-3 could enhance both basal as well as hyperactivated Akt-mTOR signaling. However, this relationship was not observed in a subset of cells, including A2780 ovarian carcinoma cells, wherein we recently showed that PRL-3 could promote cell proliferation by inducing autophagy^23^. A possible explanation to interpret this could be that PRL-3 activates autophagy and mTOR independently in different cell lines. Similar findings have been reported elsewhere, for example, in luteal cells, activation of ERK1/2 was sufficient for autophagy induction without any differential regulation of mTOR activity^49^.

PRL-3 activates PI3K/Akt signalling via various pathways, including downregulation of phosphatase and tensin homologue (PTEN)^20^ and hyperactivation of receptor tyrosine kinases^17^. Herein, we characterise mTOR as a novel downstream target of PRL-3 mainly via enhanced PI3K/Akt signalling. Surprisingly, PRL-3 also promoted mTOR activation via a parallel increase in Rag GTPase binding and lysosomal mTORC1 accumulation. Such PRL-3-driven Rag-GTP accumulation – a principal cause of mTORC1 recruitment to lysosomes – occurred in an Akt-independent manner, thereby constituting a novel mechanism of mTORC1 regulation distinct from previous mechanistic reports of upstream PI3K/Akt activation by PRL-3. This yet-characterized, Akt-independent capacity for PRL-3 to activate Rag GTPases might occur via the regulation of Rag activity via the Ragulator complex which, by promoting Rag-GTP formation, has been shown to be necessary for targeting mTORC1 to lysosomes^13^,^14^. Based on our observations, we propose that PRL-3 functions as a unique mTOR regulator in that it both 1) activates Rheb via Akt-TSC2 signaling and, in parallel, 2) promotes recruitment of mTORC1 to Rheb-resident lysosomes via interaction with Rag proteins, leading to sustained, efficient mTORC1 activation under basal and stress conditions (Fig. 7). It should be noted that in the comparative studies for acute amino acid starvation, we used a nonphysiological condition (incubation in serum-free EBSS) which – in addition to a lack of amino acids, also lacks serum, vitamins, and harbours a different salt composition from serum-supplemented RPMI-1640 – might affect other signaling pathways than amino acid starvation alone would^50^.

PRL-3-driven mTOR activation, in turn, could promote the metastatic development of cancers in at least two ways: increased motility and increased invasiveness. Both these pro-metastatic characteristics could be effectively inhibited by treatment with rapamycin, which selectivity inhibits mTORC1 but not mTORC2. Because of the importance of mTORC1 in protein translation and cell growth, PRL-3 likely exploits mTOR to upregulate the production of proteins involved in motile and invasive behaviour, especially under stress conditions. Indeed, PRL-3 overexpression resulted in a mTORC1-dependent increase in production of oncogenic MMP-2, a collagenase involved in the invasion and metastasis cascade and a marker of poor prognosis in multiple cancers^51^,^52^. Although a previous report indicates the involvement of integrin beta-1-ERK signalling in PRL-3-mediated upregulation of MMP-2^37^, here we found that PRL-3-induced MMP-2 secretion was sensitive to rapamycin-mediated mTORC1 inhibition. A possible reconciliation for these observations is that cross-activation between the Ras-MAPK and PI3K-mTORC1 pathways might co-regulate MMP-2 expression to promote tumour invasiveness. Indeed, activation of the RAS-ERK pathway has been shown to lead to mTORC1 activity by increased ERK and RSK signalling to the TSC complex^53^. We hypothesise that by endowing tumour cells with the ability to disseminate from unfavourable microenvironments (such as limited nutrient availability and/or limited oxygen) in search of more favourable conditions, PRL-3 provides a strategic survival advantage to tumour cells via increased PI3K/Akt/mTOR-dependent MMP production and invasiveness.

For decades, mTOR has emerged as an attractive target of anticancer therapy. Our data demonstrate an oncogenic role of PRL-3 on mTOR activation *in vitro* and *in vivo*, especially under oxygen- and nutrient-deprived conditions. Moreover, PRL-3 achieves this unconventionally via regulation of the two independent arms of mTORC1 activation, PI3K/Akt-driven Rheb-GTP accumulation, as well as enhanced Rag GTPase-mediated mTORC1 recruitment to endomembranes for Rheb-mediated activation. Such pleiotropic regulation by PRL-3 in driving cancer progression cements the urgent need to discover novel agents^54^ targeting this oncogene for better clinical outcomes.

## Materials and Methods

### Cells and treatments

Human colon cancer cell lines (HCT116, HCT15, LoVo, SW620, DLD-1), human cervical cancer cells (HeLa), human melanoma cells (G361), and human breast cancer cells (MCF7) were purchased from ATCC (Manassas, VA, USA). Human ovarian cancer cells (A2780) were purchased from ECACC (Salisbury, England, United Kingdom). Cell lines expressing EGFP (Vec), EGFP-PRL-3 (PRL-3), or EGFP-PRL-3 C104S (PRL-3 C104S) were generated by transfection of cells with pEGFP-C1 vector (Clontech, Palo Alto, CA, USA), pEGFP-C1-PRL-3 or pEGFP-C1-PRL-3 (C104S) constructs, respectively, using Lipofectamine 2000 (Life Technologies, Carlsbad, CA, USA), followed by G-418 selection (1 mg/mL) for 2 weeks. HCT116 cells stably expressing scrambled or PRL-3-targeting shNA constructs (Origene Rockville, MD, USA) were generated by puromycin selection (1 μg/mL) for 2 weeks. Cells were grown in RPMI-1640 medium supplemented with 10% FBS (HyClone, Carlsbad, CA, USA) and 1% (v/v) penicillin/streptomycin in a humidified incubator at 37 °C with 5% CO_2_. For serum depletion (serum-free; SF) experiments, cells were washed twice in PBS and changed to RPMI-1640 medium without FBS, supplemented with 1% (v/v) penicillin/streptomycin. For hypoxia experiments, cells were placed in a GasPak EZ Gas Pouch (BD Biosciences, Sparks, MD, USA) containing ≤1% O_2_. For amino acid starvation (AA-) experiments, cells were washed twice in PBS, once with Earle’s Balanced Salt Solution (EBSS), and finally incubated with EBSS medium for 1 h at 37 °C with 5% CO_2_. It should be noted that compared to RPMI-1640, EBSS not only lacks amino acids, but also lacks vitamins and has a different salt composition. For conditioned medium analysis, cells were cultured in RPMI-1640 medium without FBS for 24 h and medium was collected and condensed using centrifugal concentrators. Where indicated, cells were treated with DMSO-solubilised rapamycin (100 nM final; LC Laboratories, Woburn, MA, USA), DMSO-solubilized Akt Inhibitor VIII (5 μM final; Santa Cruz Biotechnology, Paso Robles, CA, USA), or DMSO alone (0.1% final).

### Antibodies, plasmids, and siRNA transfection

Antibodies against mTOR (#2983), p-mTOR S2448 (#2971), Akt (#4691), p-Akt S473 (#4060), raptor (#2280), 4E-BP1 (#9644), p-4E-BP1 T37/46 (#2855), p-p70S6K T389 (#9234), TSC2 (#4308), and p-TSC2 S939 (#3615) were purchased from Cell Signaling Technologies (Danvers, MA, USA). Anti-p70S6K (#611260) antibody was purchased from BD Biosciences (San Jose, CA, USA). Anti-LAMP2 (#ab25631) antibody was purchased from Abcam (Cambridge, England, UK). Antibodies against GST (#sc-138) and GFP (sc-9996) were purchased from Santa Cruz Biotechnology (Dallas, TX, USA). HRP-conjugated sheep anti-mouse (#515-035-062) and goat anti-rabbit (#111-035-045) antibodies were purchased from Jackson ImmunoResearch Laboratories Inc. (West Grove, PA, USA), whilst AlexaFluor568-conjugated goat anti-mouse (#A-11004) and AlexaFluor633-conjugated goat anti-rabbit (#A-21071) antibodies were from Life Technologies (Grand Island, NY, USA). Generation of anti-PRL3 antibody^55^ and PRL-3-targeting shRNA plasmids^17^ have been reported previously. The pRK5-HA-GST-RagB-WT (#19301), pRK5-HA-GST-RagC-WT (#19304), pRK5-HA-GST-RagD-WT (#19307), pRK5-HA GST RagB 54L (#19302), and pRK5-HA GST RagD 121L (#19309) constructs were gifts from David Sabatini and sourced from Addgene (Cambridge, MA, USA). Expression vectors encoding PRL-3–directed (5′-TTCTCGGCACCTTAAATTATT-3′) or non-targeting scrambled shRNA sequences were purchased from OriGene (Rockville, MD, USA). Human *AKT*-targeting (catalogue number 6211S) and control siRNA were from Cell Signaling Technologies. siRNA (100 nM final) was transiently transfected using jetPRIME (Polyplus-transfection SA, Illkirch, France) following the manufacturer’s recommended protocol.

### Western blot analysis

Western blots were performed as described^55^. Briefly, cells were washed with ice-cold PBS and lysed in RIPA buffer containing a cocktail of EDTA-free protease inhibitors (Roche, Mannheim, Germany) and phosphatase inhibitors (Nacalai Tesque, Kyoto, Japan). Lysates were clarified by centrifugation (16,000 × g, 10 min) and subjected to western blotting with indicated primary antibodies at 1:1,000 dilutions. Species-specific secondary antibodies were used at 1:2,000 dilutions. Protein-antibody conjugates were visualized using a chemiluminescent detection kit (Thermo Scientific, Waltham, MA, USA), and quantification of band intensities was done using ImageJ software. The ratio of phosphorylated/total protein was calculated and normalized as described in the figure legends.

### Immunofluorescence analysis

Immunofluorescence analysis was carried out as previously described^55^. Briefly, cells grown on glass coverslips were subjected to various treatments as indicated before rinsing with PBS and fixation for 15 min with 4% paraformaldehyde. After rinsing twice with PBS, cells were blocked for 1 h in 5% BSA blocking buffer containing 0.1% Triton-X 100, and incubated with mouse anti-LAMP2 (1:100) and rabbit anti-mTOR (1:100) antibodies overnight at 4 °C. Coverslips were then rinsed three times with PBS and subsequently incubated with secondary antibodies (1:200) for 1 h at RT followed by a further three washes in PBS. Coverslips were mounted on glass slides using DAPI-containing mounting media and analysed using an LSM700 confocal microscope (Carl Zeiss AG, Jena, Germany). The Manders coefficient (colocalization score) for each image set was calculated using the Colocalization tool within ZEN software (Carl Zeiss MicroImaging, Jena, Germany).

### *In vitro* Rag GTPase binding assay

GST-RagB and GST-RagC vectors were co-transfected into HCT116-EGFP or HCT116-EGFP-PRL-3 stable cells. The next day, culture media was replaced to incubate cells under normoxia (24 h), hypoxia (24 h), serum-free (24 h), or amino acid-free (1 h) conditions. Lysates were harvested, clarified by high speed centrifugation (14 000 × *g*, 15 min), and incubated with glutathione beads overnight at 4 °C with rotation. The beads were washed three times with lysis buffer and finally eluted with reduced glutathione (25 mM). Elutes were subjected to western blotting with the indicated antibodies.

### Rheb activation assay

Analysis of intracellular Rheb-GTP levels was done using a Rheb activation assay kit (Abcam), according to the manufacturer’s instructions. Briefly, 2.5 × 10^6^ cells, grown under the indicated culture conditions, were washed with ice-cold PBS and lysed in kit-supplied lysis buffer containing a cocktail of EDTA-free protease inhibitors (Roche). Lysates were clarified by centrifugation (16,000 × g, 10 min) before equivalent amounts of lysates (2 mg) were subjected to immunoprecipitation with a configuration-specific monoclonal antibody that specifically recognizes RheB-GTP, but not RheB-GDP. Western blotting and densitometry analysis was subsequently used to characterize the proportion of active Rheb (as reflected by the Rheb-GTP/Rheb ratio) present in cellular extracts.

### Wound healing assay

3.5 × 10^4^ HCT116-EGFP or HCT116-EGFP-PRL-3 cells were seeded into μ-dishes (Ibidi, Martinsried, Germany) and cultured under normal conditions until they reached confluence. Subsequently, inserts were removed to yield standardized 500 μm-wide gaps. ‘Wounded’ cell monolayers were washed in PBS and subsequently cultured in low-serum medium (0.5% FBS) with 100 nM rapamycin or 0.1% DMSO for 48 h. Images were acquired sequentially every 24 h using an Axiovert 200M inverted microscope (Carl Zeiss AG).

### Invasion assay

1 × 10^4^ HCT116-EGFP or HCT116-EGFP-PRL-3 cells were suspended in complete medium containing 100 nM rapamycin or 0.1% DMSO and seeded into BioCoat Matrigel invasion chambers with 8.0 μm PET membranes (Corning, MA, USA). After incubation for 24 h at 37 °C, non-invading cells were removed from the upper surface of the membrane by a cotton swab, and membrane inserts were washed three times with PBS and fixed in 4% paraformaldehyde for 15 min. Subsequently, membranes were cut, mounted inverted on glass slides, and observed for EGFP fluorescence under an LSM700 confocal microscope (Carl Zeiss AG). The numbers of invaded cells in four ramdomly-chosen fields were counted. Data were presented as mean ± SD of cell number/field and statistically analyzed using the Student’s *t*-test.

### Human studies

Human gastric tissue samples were obtained with patient consent from the National University Hospital-National University of Singapore (NUH-NUS) Tissue Repository. Experimental procedures were approved by the Institutional Review Board (IRB) of NUH-NUS for research use and conducted in accordance with approved guidelines and regulations.

### Analysis of mouse mammary tissues

For the isolation of mammary tissue lysates, the uppermost pair of mammary glands from wild-type FVB/N mice or MMTV-PyMT mice (at 6, 9, 12, and 15 weeks) were surgically removed, rinsed in PBS, and homogenised in RIPA lysis buffer using a Polytron homogenizer (Luzern, Switzerland) prior to western blot analysis. All animal studies were approved by an Institutional Animal Care and Use Committee (IACUC) and were performed in accordance with approved guidelines and regulations of the Biological Resource Centre, A*STAR, Singapore.

### Gene expression analysis of colon cancer patient dataset

Statistical analyses of the colon cancer patient dataset GSE40967 (*n* = 566) were performed using SPSS 19.0 software (IBM, NY, USA). Correlation between PRL-3 and MMP-2, MMP-7, or MMP-9 gene expression was analysed by Spearman Correlation. The association between PRL-3 expression and relapse-free survival (RFS) was analysed by Kaplan-Meier analysis.

## Additional Information

**How to cite this article**: Ye, Z. *et al.* PRL-3 activates mTORC1 in Cancer Progression. *Sci. Rep.* **5**, 17046; doi: 10.1038/srep17046 (2015).

## Supplementary Material

Supplementary Information
