# Supplementary material for: PRL-3 activates mTORC1 in Cancer Progression
Source: Sci Rep. 2015 Nov 24;5:17046. doi: 10.1038/srep17046 (PMC4657013; doi:10.1038/srep17046)

Supplementary Information

Supplement to submitted manuscript titled:

PRL-3 activates mTORC1 in Cancer Progression

Zu Ye[1,2†], Abdul Qader Omer Al-aidaroos[1†], Jung Eun Park[1†], Hiu Fung Yuen[1], Shu Dong Zhang[3], Abhishek Gupta[1], Youbin Lin[1], Han-Ming Shen[2]*, and Qi Zeng[1,4]*

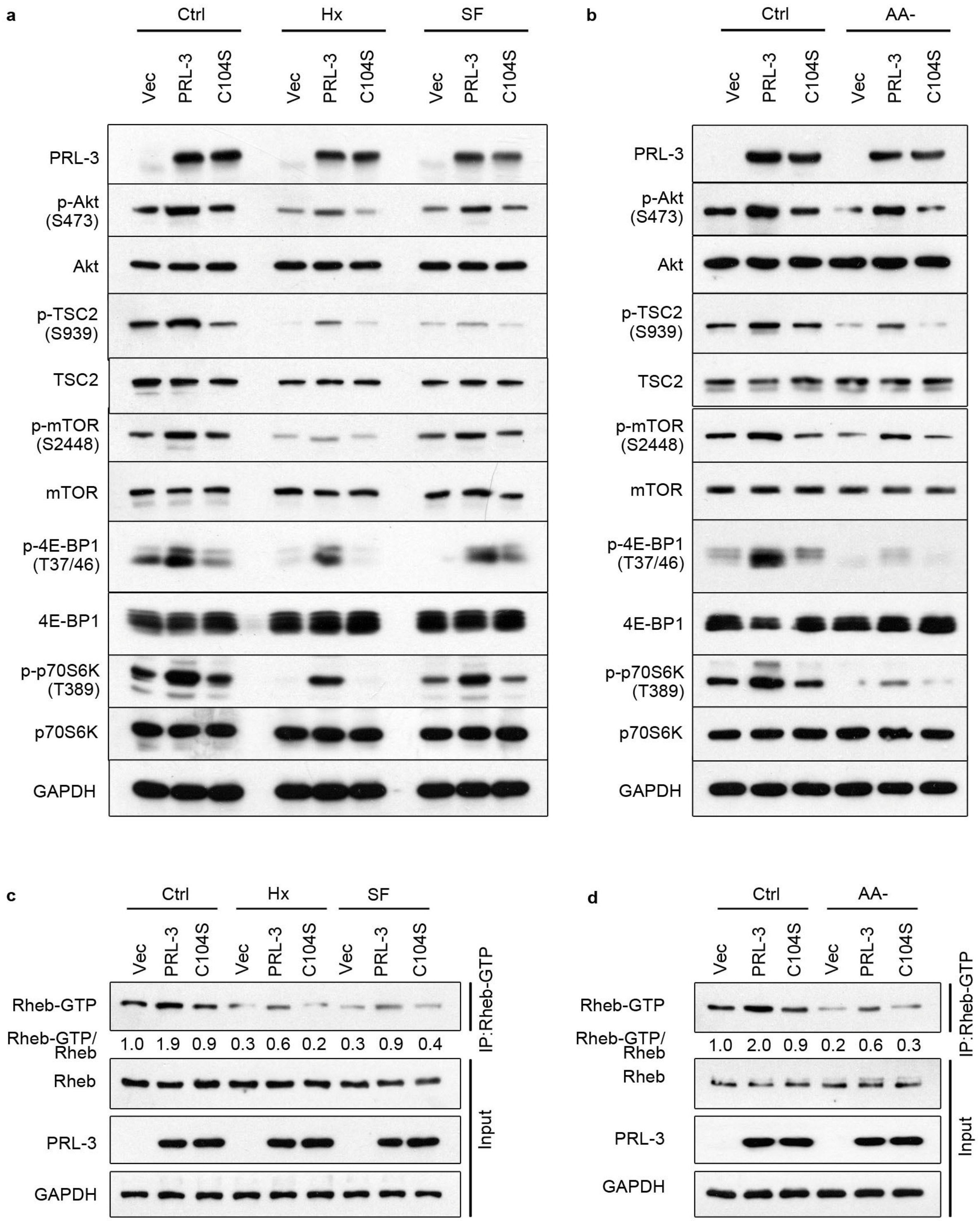

Supplementary Figure 1. Overexpression of wild-type PRL-3 increases Akt-mTOR activity in HeLa cells. (a) HeLa cells overexpressing EGFP (Vec), EGFP-PRL-3 (PRL-3), or catalytic-

inactive mutant EGFP-PRL-3 C104S (C104S) were cultured for 48 h under normoxia (Ctrl), hypoxia (Hx), or serum-free (SF) conditions prior to lysis and western blot analysis with the indicated antibodies. **(b)** HeLa cells overexpressing Vec, PRL-3 or C104S were cultured for 1 h in full media under normoxia (Ctrl) or amino-acid starved (AA-) conditions, prior to analysis as in **(a)**. **(c)** Cell lysates from **(a)** were immunoprecipitated with a configuration-specific anti-Rheb-GTP antibody and analysed by immunoblotting with the indicated antibodies. The ratio of Rheb-GTP/total Rheb band densities were calculated and normalized to the Rheb-GTP/total Rheb ratio in the first lane. **(d)** Cell lysates from **(b)** were analyzed as in **(c)**.

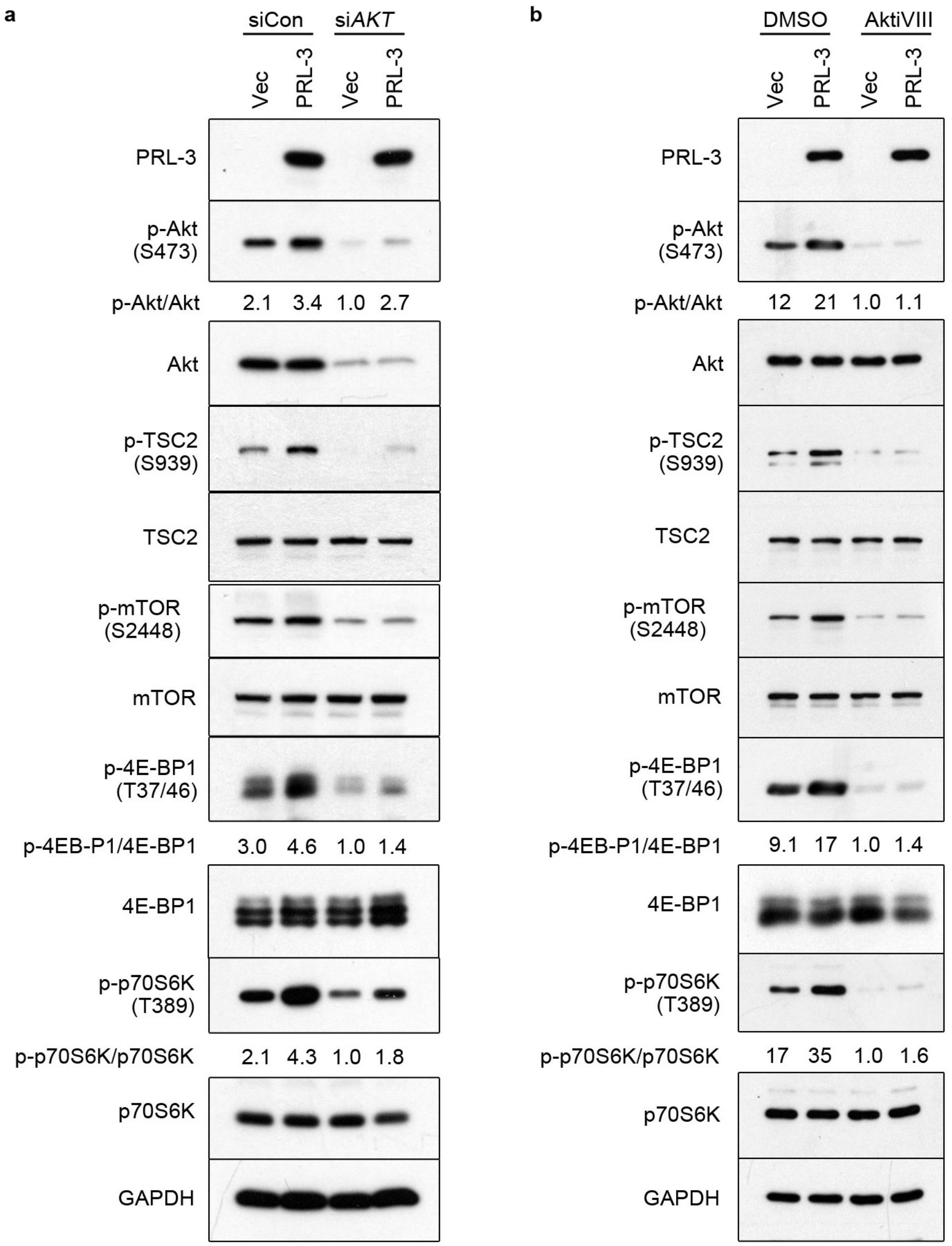

Supplementary Figure 2. Akt activity is required for PRL-3-mediated hyperactivation of mTOR signaling. (a) Akt in HeLa cells stably expressing EGFP (Vec) or EGFP-PRL-3 (PRL-3) were transiently depleted using scrambled small interfering RNA (siRNA; siCon) or AKT-targeting siRNA (si*AKT*) and cultured for 48 h before analysis of Akt-mTOR pathway activity.

The ratio of phosphorylated/total band densities for Akt, 4E-BP1, and p70S6K were calculated and normalized to their cognate phosphorylated/total protein ratio in lane 3. **(b)** Akt in HeLa Vec or PRL-3 cells was inhibited for 30 min using Akt inhibitor VIII (AktiVIII) before analysis of Akt-mTOR pathway activity as in **(a)**. The ratio of phosphorylated/total band densities for Akt, 4E-BP1, and p70S6K were calculated and normalized to their cognate phosphorylated/total protein ratio in lane 3. Full immunoblots for the cropped images presented here are provided in the Supplementary Information.

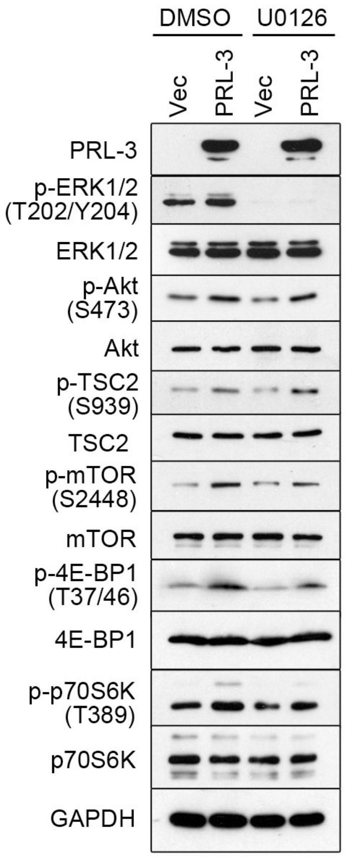

Supplementary Figure 3. PRL-3-mediated hyperactivation of mTOR signaling is ERK-independent. HCT116 cells overexpressing EGFP (Vec) or EGFP-PRL-3 (PRL-3) were cultured for 24 h in the presence of 0.1% DMSO or 10 μM U0126 prior to lysis and western blot analysis with the indicated antibodies. Full immunoblots for the cropped images presented here are provided in the Supplementary Information.

Full unedited gels for Figure 1

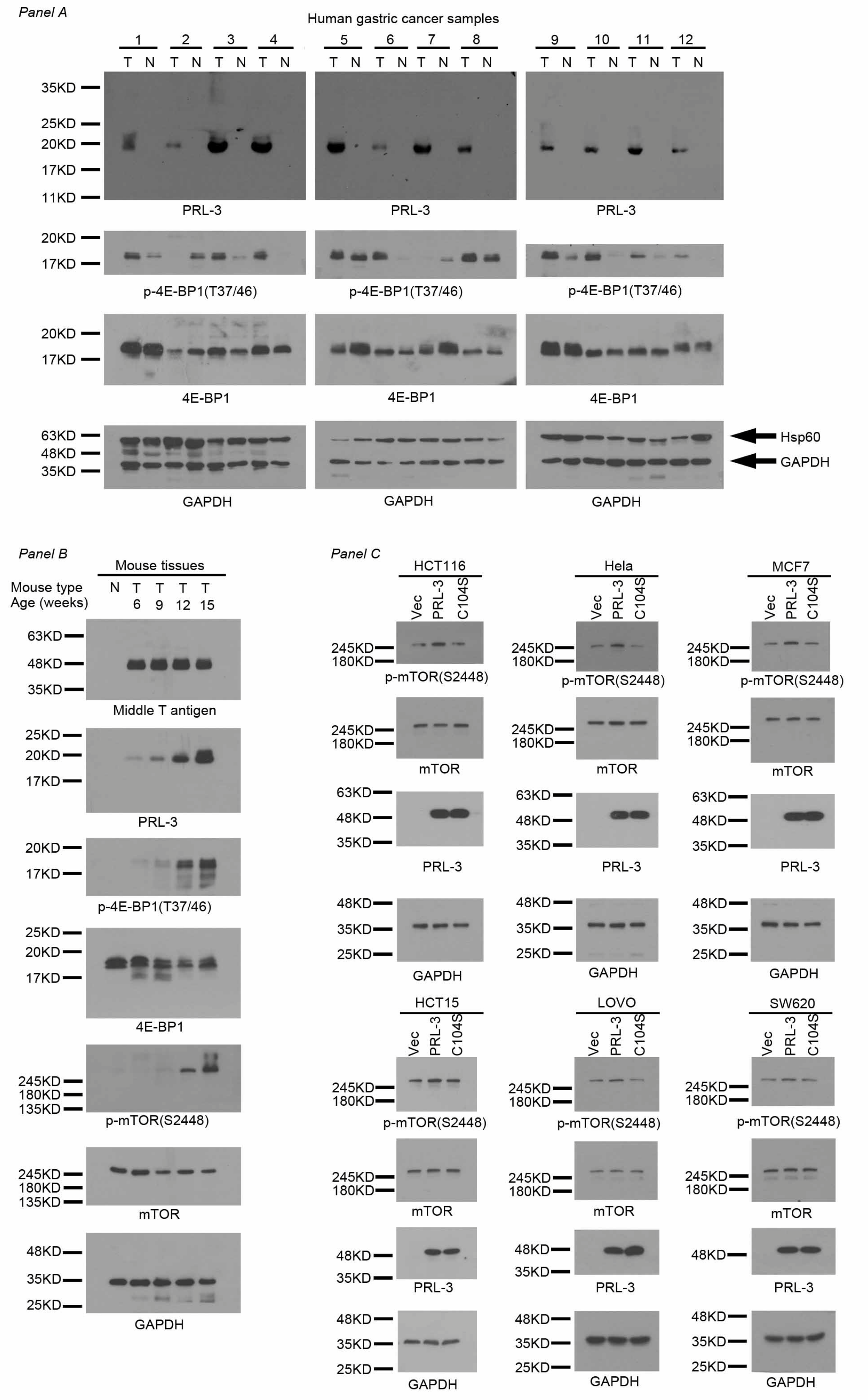

Full unedited gels for Figure 1

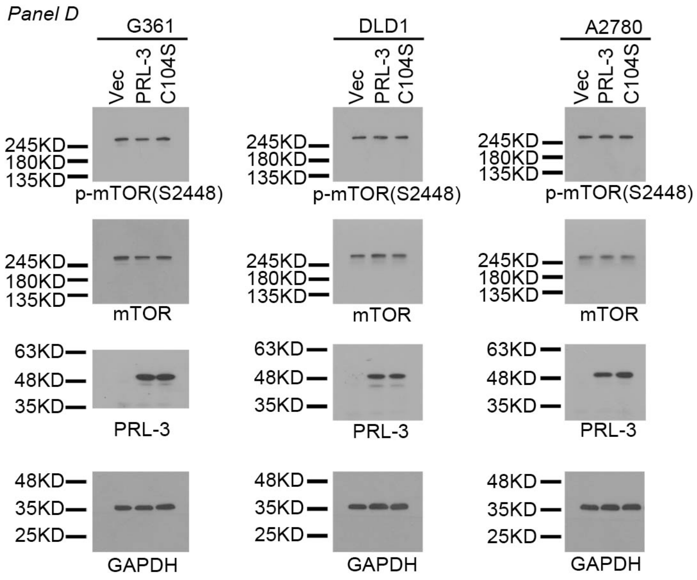

Full unedited gels for Figure 2

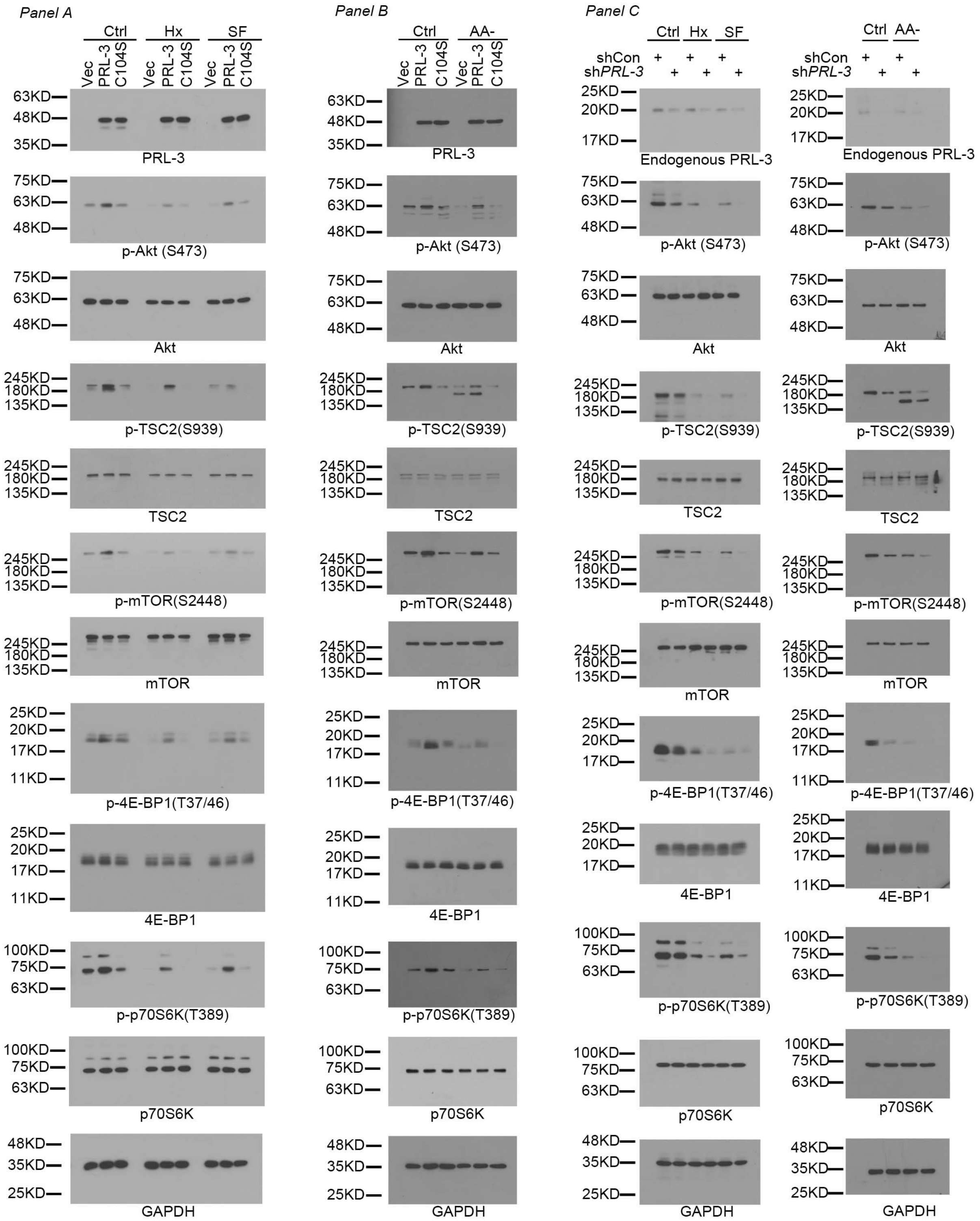

Full unedited gels for Figure 2

Panel D

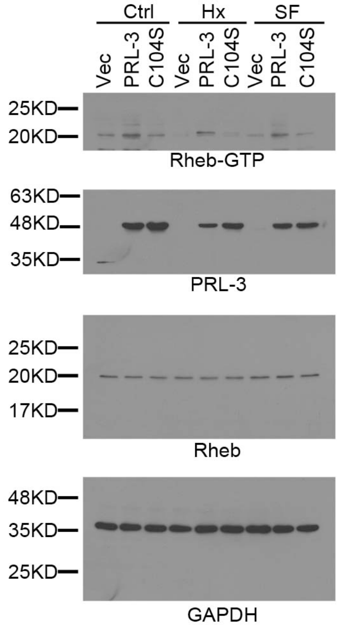

Panel E

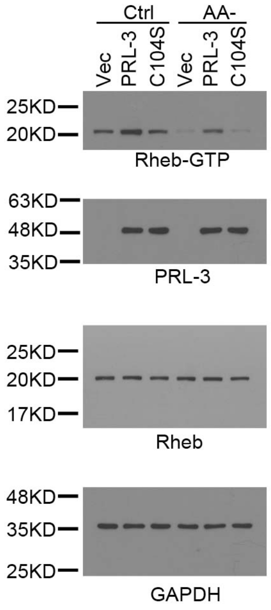

Full unedited gels for Figure 3

Panel A

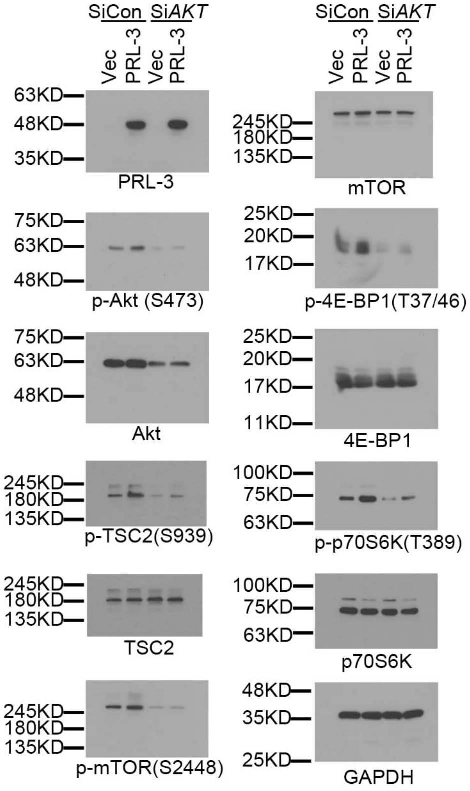

Panel B

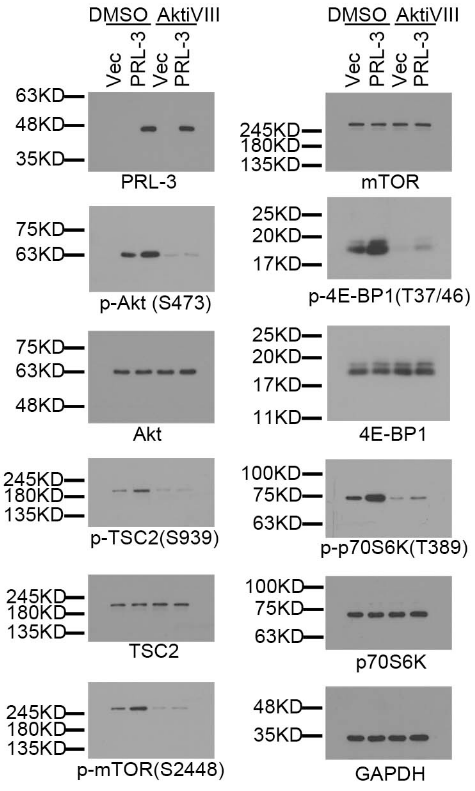

Full unedited gels for Figure 5

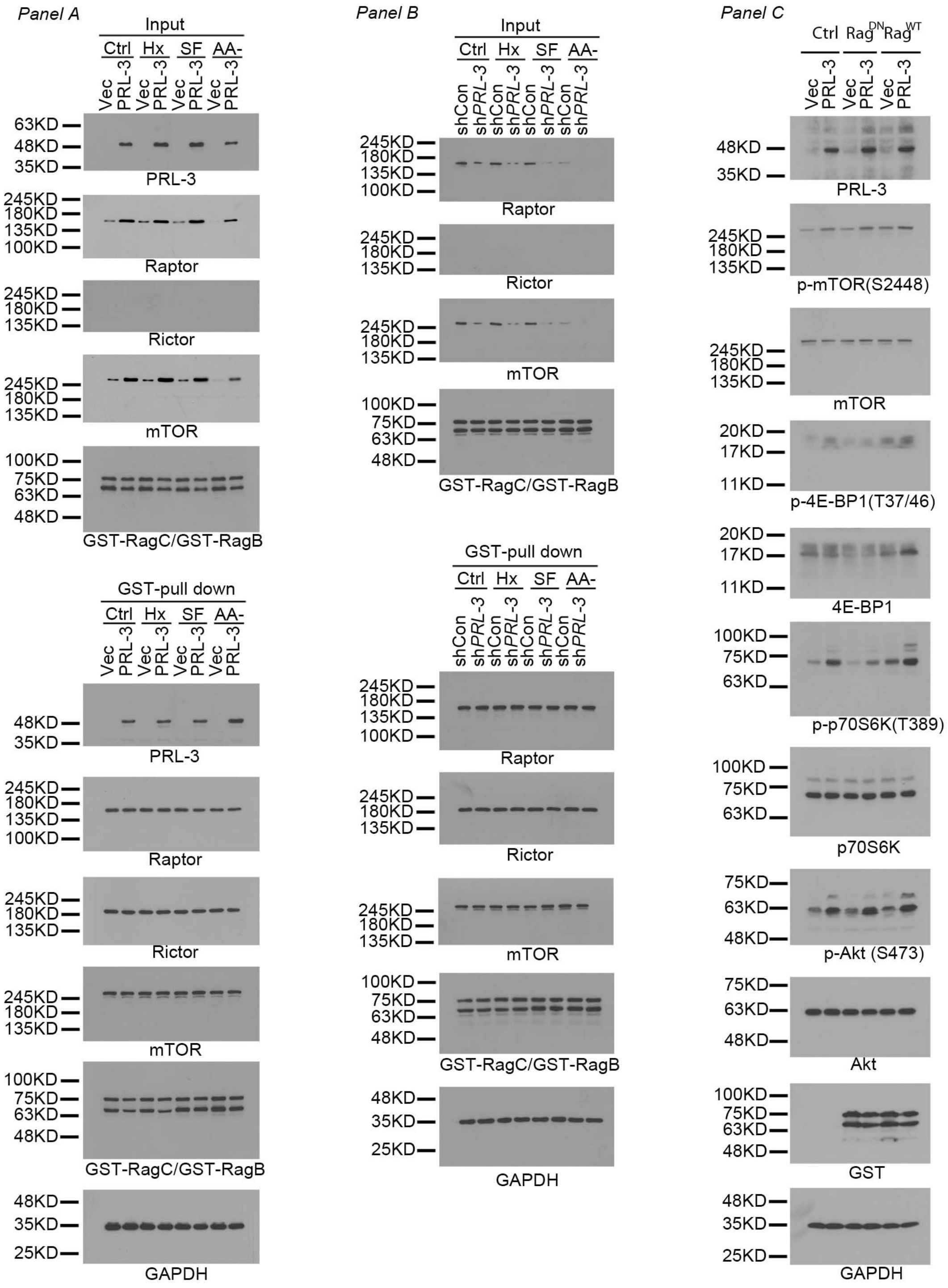

Full unedited gels for Figure 6

Panel C

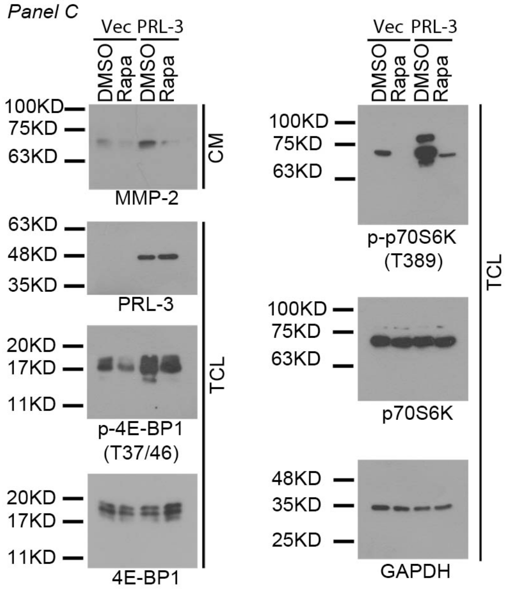

Full unedited gels for Supplemental Figure 1

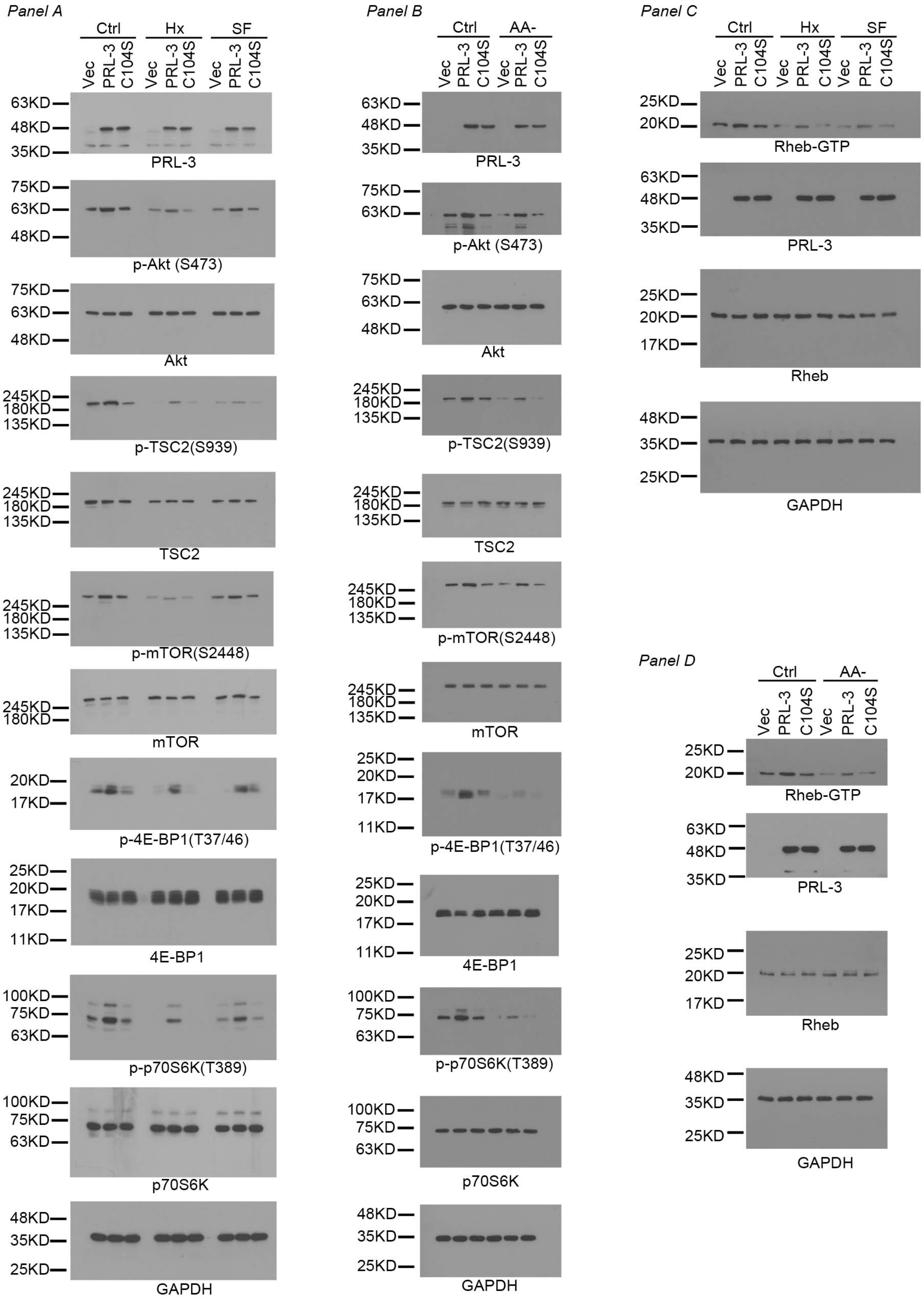

Full unedited gels for Supplemental Figure 2

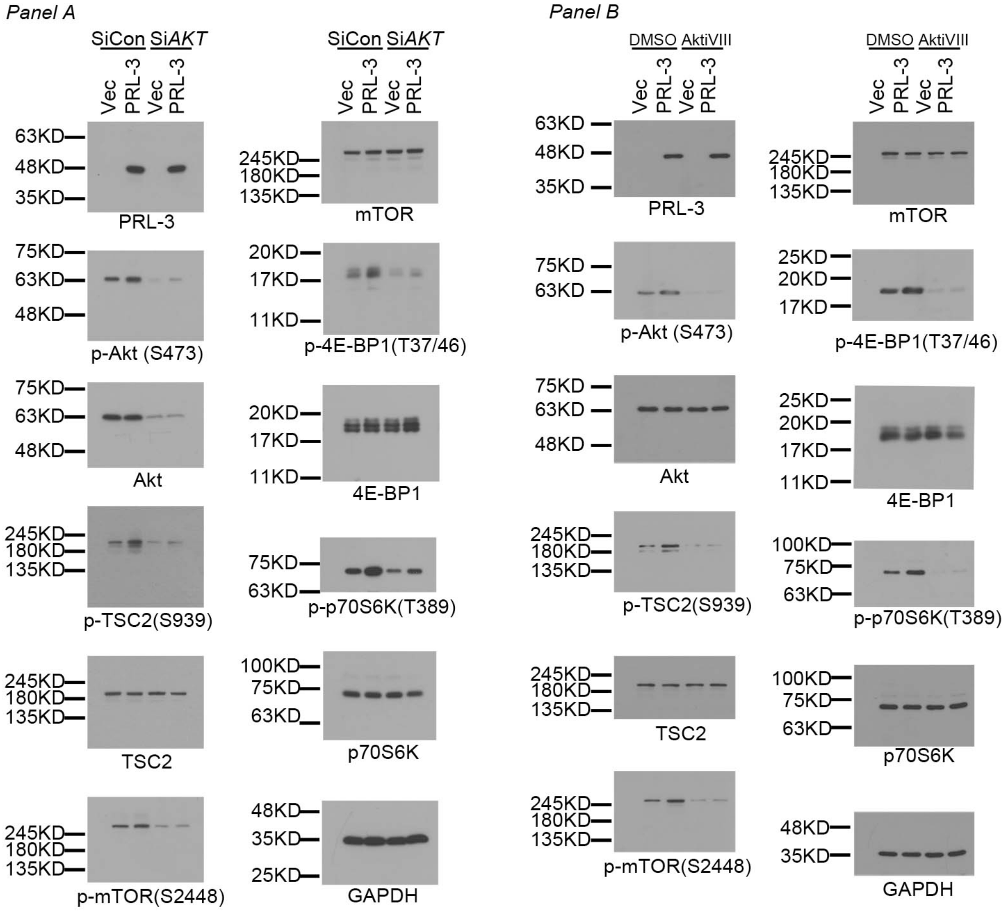

Full unedited gels for Supplemental Figure 3

Panel B

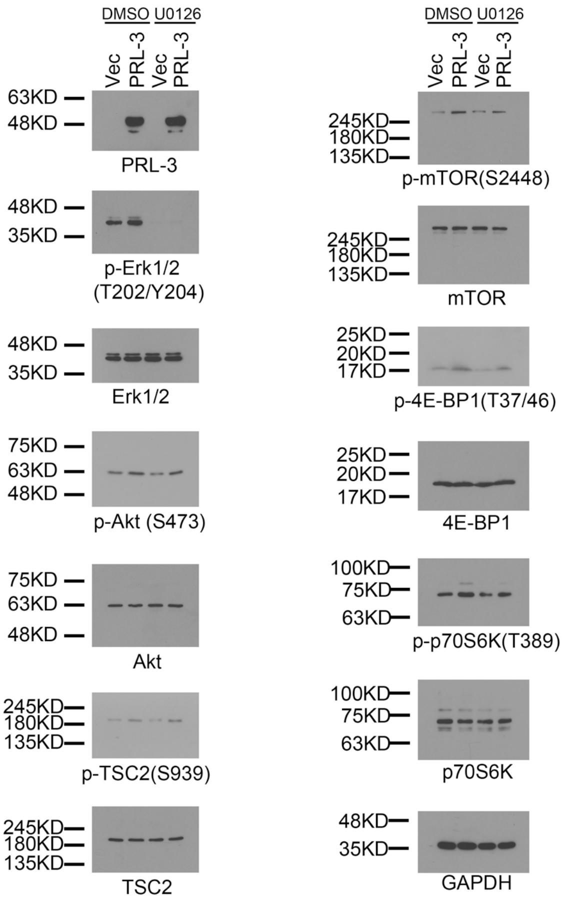

Supplement: Supplementary Information [file srep17046-s1.pdf]
